# Effect of Heavy Metals Pollution on Soil Microbial Diversity and Bermudagrass Genetic Variation

**DOI:** 10.3389/fpls.2016.00755

**Published:** 2016-05-31

**Authors:** Yan Xie, Jibiao Fan, Weixi Zhu, Erick Amombo, Yanhong Lou, Liang Chen, Jinmin Fu

**Affiliations:** ^1^Key Laboratory of Plant Germplasm Enhancement and Specialty Agriculture, Wuhan Botanical Garden, Chinese Academy of SciencesWuhan, China; ^2^Graduate University of Chinese Academy of SciencesBeijing, China; ^3^College of Resources and Environment, Shandong Agricultural UniversityTai’an, China

**Keywords:** bermudagrass, cadmium, microorganism diversity, Cd-tolerance, genetic diversity

## Abstract

Heavy metal pollution is a serious global environmental problem as it adversely affects plant growth and genetic variation. It also alters the composition and activity of soil microbial communities. The objectives of this study were to determine the soil microbial diversity, bermudagrass genetic variation in Cd contaminated or uncontaminated soils from Hunan province of China, and to evaluate Cd-tolerance of bermudagrass at different soils. The Biolog method, hydroponic experiments and simple sequence repeat markers were used to assess the functional diversity of microorganisms, Cd-tolerance and the genetic diversity of bermudagrass, respectively. Four of the sampling sites were heavily contaminated with heavy metals. The total bioactivity, richness, and microbial diversity decreased with increasing concentration of heavy metal. The hydroponic experiment revealed that bermudagrass populations collected from polluted sites have evolved, encompassing the feature of a higher resistance to Cd toxicity. Higher genetic diversity was observed to be more in contaminated populations than in uncontaminated populations. Heavy metal pollution can result in adverse effects on plant growth, soil microbial diversity and activity, and apparently has a stronger impact on the genetic structure. The results of this study provide new insights and a background to produce a genetic description of populations in a species that is suitable for use in phytoremediation practices.

## Introduction

Soil pollution by heavy metals is a critical global environmental problem. Experts have estimated that more than 20 million hectares of farmland in China have been contaminated, accounting for 20% of the total landmass (Wei and Chen, 2001). Cd is considered to be one of the most phytotoxic metal pollutants due to its high mobility (especially in soil with low CEC and acidic pH), bioaccumulation in lower organisms and its easily being transferred to higher trophic levels in the food chain (Lin and Aarts, 2012). The toxicity of Cd pollution and its physical disturbance can influence plant survival, reproductive success and migration (Deng et al., 2007). Plants are sedentary; they lack the ability to move actively to evade contaminated environment. Therefore, their only chance to survive in unfavorable conditions is the mobilization of defense mechanisms, and evolution of tolerate genotype (Chmielowska-Bąk et al., 2014).

Some plants are genetically adapted to grow and reproduce in soils contaminated with heavy metals. Nonetheless, plants that grow in uncontaminated soils have evolved tolerant ecotypes able to survive in toxic environments (Kruckeberg, 1967). Hence, plant populations growing at contaminated sites were often genetically distinct from the populations of the same species in adjacent non-contaminated sites (Assunção et al., 2003). Such metal-tolerant populations provide a typical example of microevolution (Macnair, 1993). Previous studies investigating the population genetics of this phenomenon indicated that the evolution of heavy metal-tolerant ecotypes occur at an unexpected frequency, thereby maintaining a high polymorphism. This phenomenon appeared to be the least variable parameter when compared to non-tolerant populations despite the founder effect and selection (Gartside and McNeilly, 1974; Lefèbvre and Vernet, 1990; Mengoni et al., 2000). However, inter-population variation showed no relation to the geographical origin or heavy metal tolerance (Lefèbvre and Vernet, 1990). By the use of suitable molecular markers it was possible to reveal the relationship between heavy metals tolerance and the genetic variability. The molecular markers linked to metal tolerance in natural populations could be necessary for improving the phytoremediation techniques and elucidation of the molecular basis of tolerance (Mengoni et al., 2000). Among the molecular markers, simple sequence repeat (SSR) markers have been exploited extensively in studies on the genetic variability in many plants due to its locus specificity, high polymorphism, and reproducibility. However, it has not been applied to study a metal-tolerant plant population.

Soil microorganisms, both free-living and symbiotic soil microbes in the rhizosphere of plants growing on metal contaminated soils, can increase plant biomass production and enhancing phytoremediation process. However, heavy metals affect the growth, morphology, and metabolism of soil microorganisms, through functional disturbance, protein denaturation or the destruction of the integrity of cell membranes (Leita et al., 1995). Soil microorganisms are essential in the decomposition of soil organic matter; any decrease in the microbial diversity or abundance may adversely affect nutrient absorption from the soil for plant (Giller et al., 1998a). The elevated levels of heavy metals in soils had significant impacts on the population size and overall activity of the soil microbial communities. Several studies, depending on the isolation-based techniques used, have revealed that heavy metal contamination gave rise to shifts in microbial populations (Gingell et al., 1976; Barkay et al., 1985; Roane and Kellogg, 1996). However, isolation-based techniques are limited because they only represent a small component of the microbial community. This limitation could be attributable to the fact that only a small percentage of soil microbes are culturable. A relatively improved procedure that may be useful in evaluating changes in microbial community structure is the determination of the metabolic profile of a particular system via the Biolog procedure (Garland, 1997). Biolog has been effective in characterizing the functional capability of soil organisms to utilize specific carbon substrates (Garland and Mills, 1991; Garland, 1996), and also has been widely used in assessing the functional diversity, associated with the microbial community in soil samples from farmland and grassland ecosystems (Sarathchandra et al., 2001; Rutgers et al., 2016).

Bermudagrass [*Cynodon dactylon* (L.) Pers.] is economically seen the most important and extensively used warm-season turfgrass. It is distributed across all continents and on most ocean islands between latitudes of approximately 45° North and South. It is highly polymorphic, with forms adapted to a range of climatic and edaphic conditions (Wu, 2011). Bermudagrass depends entirely on grazers to enter the food chain. Some previous studies and our recent investigation demonstrated that bermudagrass was the most abundant and dominant species at heavy metal-contaminated soils, especially in the south of China. This observation implies that it might have evolved tolerance to heavy metal (Shu et al., 2002; Archer and Caldwell, 2004).

The objectives of this study were to: (1) evaluate the soil microbial community’s diversity of heavy-metal-contaminated soils using the Biolog-Ecoplate method; (2) investigate the Cd-tolerance in four metallicolous and non-metallicolous populations of bermudagrass based on the integrated tolerance index calculated with fuzzy subordinate functions (She et al., 2011; Xie et al., 2014b); and (3) determine the genetic diversity of these populations using the SSR method.

## Materials and Methods

### Sample Collection

The sampling sites were in Hunan Province, Central China, and each consisted of two separate field sites including polluted and un-polluted (control) site. The polluted areas were four separate field sites, the first was Liuyang (113°21′N, 28°01′E, in the vicinity of a chemical factory in Liuyang City), and the second was Xiangtan (112°57′N, 27°52′E, near a chemical factory in Xiangtan City). The third site was Zhuzhou (113°04′N, 27°52′E, adjacent to the land between a chemical factory and a cement plant in Zhuzhou City) and, the final one was Yueyang (113°26′N, 29°18′E, around lead-zinc mine factory of Yueyang City). Sites selected as controls (i.e., Cd un-polluted soil) were 50 km away from those four different polluted sites mentioned above, respectively. The first three samples were of identical texture (red soil) and had similar organic matter content (8 to 14 g kg^-1^) and pH (5.4 to 6.2). The soil type in the Yueyang area was sand loam. Major environmental conditions such as temperature, water content, etc., were presumably similar except for the heavy metal content.

Bermudagrass, a common and dominant species, was collected from each location according to the topography. Associated soil samples from the study sites were also collected from the root zone of bermudagrass. There were five replicates at a 50 m space from each location. Each soil sample was put into polyethylene bags and stored until transportation to the laboratory. Soil samples were sieved with stainless steel mesh (mesh size, 2 mm). Plant debris and soil animals were removed, and then the remainder stored at -20°C until analysis. The roots, stolons, stems, and leaves of bermudagrass were harvested separately and immersed in 10 mM Na_2_EDTA for 30 min and then rinsed thoroughly with distilled water. The samples were oven-dried at 75°C to constant weight and grinded into fine powder. The total concentration of heavy metals (Cd, Pb, and Zn) in the soils and plants was analyzed by flame atomic absorption spectrophotometry (AAS, Z-5300) by digesting 100 mg of soil in a mixture of HNO_3_ and HClO_4_ (4:1, v/v).

### Functional Diversity of Microorganisms

The functional diversity of soil microorganism was analyzed using Biolog-Ecoplate, comprising of different substrates present in soils. The extraction of soil samples was by shaking 10 g of soil with 100 mL 0.1 M Tris buffer (pH 7.5) for 10 min. After shaking, samples were centrifuged for 10 min at 2,600 *g*. The supernatant was diluted, and 150 μL from the 10^-3^ dilutions were inoculated into Biolog-Ecoplates (Biolog, Hayward, CA, USA). Each of the 96-well plates contained 31 different carbon sources separately and a blank well, each replicated three times. The plates were incubated at 28°C and substrate utilization was monitored by measuring absorbance using an automated plate reader (ELX808, Labsystems, Helsinki, Finland) at 590 nm every 12 h for 168 h and the data were collected using Microlog 4.01 software (Biolog). Additionally, the average well-color development (AWCD) was also calculated by averaging the final absorbance values for all substrate wells, as described by Garland (1997). Using the AWCD values of 31 carbon sources for each sample prior to statistical analysis eliminates variable responses that may occur as a function of differences in initial cell densities (Garland and Mills, 1991; Garland, 1997; Classen et al., 2003). The Shannon index (H) was calculated using the equation: $H=-\Sigma_{i=1}^{n}p_{i}$ In p_i_ where *p*_i_ is the proportion of the corrected absorbance value of each well to the sum of absorbance values of all wells and n is the total number of carbon sources (Garland, 1997). Statistical analysis of the 72 h AWCD and *H* values were performed because the best resolution occurred at the shortest incubation time of 72 h (Lewis et al., 2010). Furthermore, the 31 carbon sources in the plates can be divided into four categories, including amino acid and its derivatives (AD), sugar and its derivatives (SD), fatty acid and its derivatives (FD), and secondary metabolites (SM) (Liu et al., 2012).

### Effects of Cd Polluted on the Cd-Tolerance in Bermudagrass

A total of 40 bermudagrass accessions were collected from the four sites mentioned above. Each accession was allowed to propagate vegetatively in a plastic pot filled with sand. Plants were fertilized weekly with half-strength Hoagland’s solution (Hoagland and Arnon, 1950). The grasses were irrigated three times, and hand clipped at a height of 10 cm every week. All the pots were placed in a greenhouse for 40 days to allow the establishment of roots and shoots with the temperature set at 30/25°C (day/night, 10 h light/14 h dark). After 40 days establishment, the plants were rinsed thoroughly using distilled water and each accession was divided into two groups (four replicates each); the control (CK) and the Cd treatment (Cd). The plants were then transferred to some pre-prepared Erlenmeyer flasks (300 mL in volume) filled with 280 mL of half-strength Hoagland’s solution containing MgO_2_, to supply oxygen. The plant-flask system was sealed using plasticine and a sealing film at the crown of plants, and covered with parafilm. The flasks were wrapped with a silver paper to prevent potential algal growth and kept in the growth chambers for 2 weeks to allow the recovery of roots’ uptake capabilities and the adaptation to hydroponic culture conditions. Growth chambers conditions during experimental period were the average light intensity of 400 μmol photons m^-2^ s^-1^ on the top of bermudagrass canopy, 10 h photoperiod, and 70 ± 10% relative humidity. During weeks of adaptations, initial transpiration was measured by the difference in the flask-plant system weight within a 24 h interval.

#### Experimental Treatments

After adaptation, plants were exposed to 0 (CK, half-strength Hoagland solution) and 1.5 mM Cd (Cd, CdSO_4_^.^8/3 H_2_O dissolved in half-strength Hoagland solution), and each flask contained 0.5 mol MgO_2_ to provide additional oxygen. The containers with plants were kept in the greenhouse with the conditions described above for 3 weeks. The accessions and Cd treatment were arranged in a completely randomized design. The turf quality (TQ), chlorophyll content (Chl), transpiration rate (TR) and growth rate (GR) parameters were determined weekly. Before commencement of Cd treatment, the young leaves of each accession were collected and immediately frozen in liquid nitrogen, and then stored in a freezer at -80°C for DNA extraction.

#### Measurements

The turf quality is a particularly important trait and was assessed combined visual ratings of color intensity, leaf texture, and density. It was rated visually on a scale of zero to nine, where zero represented withered, thin, yellow or dead grass while nine represented green, dense, and uniform grass. Six, on the other hand, accounted for the minimum acceptable level according to color (percentage green leaves) and density of the grass (Turgeon, 1991).

To measure the chlorophyll content, 0.1 g of fresh leaves were cut into small pieces and placed in 15 mL tubes. Then 10 mL dimethylsulfoxide was added to the tubes and kept under dark conditions for 72 h. The absorption (OD) of samples was measured at 645 and 663 nm with a spectrophotometer (UV-2600, UNICO, Shanghai). The calculation of chlorophyll content was according to the equation described by Hiscox and Israelstam (1979).

To determine the growth rate, a sheet of filter paper was twisted into a tube resting on the Erlenmeyer flask to ensure the plant materials were cut at the same height every week. Fresh weights of clippings were recorded. The growth rate was the difference in fresh weight before and after cutting at 7-day intervals. The transpiration rate was determined using the method described by Hu et al. (2011).

Cadmium-tolerant comprehensive evaluation of all bermudagrass accessions was accessed by membership function method of fuzzy mathematics (Xie et al., 2014b).

### Molecular Marker Characterization

Total DNA was extracted and quantified as described by Xie et al. (2012). Amplification reactions were performed in a 10 μL total volume containing: 1 μL of 1 pmol μL^-1^ forward primer, 1 μL of 1 pmol μL^-1^ reverse primer, 0.6 μM MgCl_2_, 1 μL 1 × Taq buffer [with (NH_4_)_2_SO_4_], 0.2 μL of 10 mM dNTPs (Pharmacia, New York, America), 2 μL of 15 ng μL^-1^ DNA template, 0.25 μL of 1 U μL^-1^ Taq DNA polymerase and 3.95 μL ddH_2_O. A mastercycler gradient PCR machine (Eastwin, China) was employed to perform the reactions. The initial cycle was at, 94°C for 5 min followed by 14 cycles at 94°C for 20 s, 58°C for 1 min, 72°C for 30 s, 28 cycles at 94°C for 20 s, 55°C for 1 min, 72°C for 30 s. A final extension step at 72°C for 10 min was performed. A set of 77 pairs of SSR primers were provided by Dr. Yanqi Wu (at the Department of Plant and Soil Sciences, Oklahoma State University), who developed and characterized a large set of SSR markers in *Cynodon transvaalensis* from its SSR-enriched genomic libraries (Tan et al., 2014). These primers were screened with six accessions for polymorphism and reproducibility. As a result, 46 pairs of primers generated stable, polymorphic and reproducible fragments that were selected for final SSR analysis (**Supplementary Table S1**). The forward primer was 5′ end-labeled with three fluorescent dyes of different colors [HEX (green), TAM (yellow), or FAM (blue)] to facilitate the universal labeling of PCR products (**Table 1**). The amplified fragments were separated in an ABI 3730 DNA Sequencer (Applied Biosystem, Inc., Foster City, CA, USA). Alleles were recorded using GeneMarker 1.5 Software and manually checked twice for accuracy. The bands were recorded as a data matrix for the presence (1) and absence (0) of bands. Only those distinct and reproducible bands were scored.

**Table 1:** The climatic and soil conditions of polluted areas in Hunan province.

Sampling sites	Average annual temperature (°C)	Annual rainfall (mm)	Soil type	Total heavy metal concentration (mg kg^-1^ dry soil)		
				
				Zn	Pb	Cd
Liuyang	17.3	1562.0	Polluted	1017 ± 101	1058 ± 54	46 ± 4
			Un-polluted	869 ± 74	77 ± 12	2 ± 0.3
Zhuzhou	17.2	1471.0	Polluted	1339 ± 65	2032 ± 47	106 ± 24
			Un-polluted	1050 ± 35	107 ± 4	6 ± 0.4
Xiangtan	17.1	1350.0	Polluted	1008 ± 54	36 ± 2	30 ± 2
			Un-polluted	923 ± 26	ND	6 ± 1
Yueyang	17.1	1353.0	Polluted	1096 ± 65	169 ± 15	9 ± 0.4
			Un-polluted	1033 ± 77	ND	4 ± 0.6


*^a^ND means not detected. The data represent means ± standard error (SE, *n* = 5).*

### Data Analysis

The main treatment effect, correlation between phenotypic traits, and variance analysis of (ANOVA) were performed using the SPSS package (version 20.0; SPSS, Chicago, IL, USA). Significance of differences between sites and pollution categories or other parameters were assessed using two-way ANOVAs combined with Tukey tests at 0.05 level of probability (SPSS 20.0; Chicago, IL, USA).

Methods of analysis of Biolog data referred to Garland and Mills (1991). The measurements of individual substrates were corrected for background absorbance by subtracting the absorbance of the control samples. All experimental data were analyzed by one-way ANOVA. For multivariate analysis of the Biolog data the absorbance values were first transformed by division by the AWCD to avoid bias between samples with different inoculum densities (Garland, 1996). Principal component analysis (PCA) was conducted with SPSS 20.0 (Chicago, IL, USA) to evaluate the influence of metal contamination on soil microbial community.

Gene diversity and polymorphic information content (PIC) of populations were computed by the mean of pairwise differences among phenotypes of all members of the populations using the PowerMarker program (a comprehensive set of statistical methods for genetic marker data analysis developed and distributed by Jack Liu^1^). Dendrogram based on Nei’s genetic distances were generated using the unweighted pair group method with arithmetic average (UPGMA; Nei, 1972), and it was constructed using the Mega software version 1.01.

## Results

### Metal Concentrations in Soils and Plants

All the polluted soils (Liuyang, Zhuzhou, Xiangtan, and Yueyang) contained extremely high Pb and Cd concentrations, which were one to two orders of magnitude higher than those in un-polluted soils (**Table 1**). Among the polluted sites, the total Zn, Pb, and Cd concentration in Zhuzhou were higher than in the others, while the second highest values of total Cd and Pb were from Liuyang.

Bermudagrass collected from the polluted sites contained a higher level of heavy metals (Cd and Pb) than those from the un-polluted sites (**Table 2**). The Zn concentration was greater in Liuyang and Zhuzhou polluted sites as compared to the control for root, stolon, stem, and leaf, while no notable difference in Zn concentration was present between Xiangtan and Yueyang polluted sites and un-polluted sites. Heavy metal concentrations were also different among the polluted populations; the Zhuzhou population contained the highest level of Zn and Pb while the Liuyang population had the highest Cd. The lowest contents of Zn and Cd in the four contaminated populations were always found in the Xiangtan population.

**Table 2:** Heavy metal concentrations in plants from different bermudagrass populations.

Total concentrations (mg kg^-1^, DW)	Tissue	LY-polluted	LY-un-polluted	ZZ-polluted	ZZ-un-polluted	XT-polluted	XT-un-polluted	YY-polluted	YY-un-polluted
Zn	Root	3007 ± 348 a	847 ± 283 b	3047 ± 32 a	1581 ± 339 b	530 ± 80 b	658 ± 85 b	1403 ± 323 b	888 ± 125 b
	Stolon	2673 ± 288 b	1022 ± 204 bc	3818 ± 47 a	1454 ± 233 bc	591 ± 93 c	510 ± 36 c	1394 ± 365 bc	1043 ± 199 bc
	Stem	2514 ± 388 a	633 ± 76 b	2745 ± 37 a	1461 ± 306 b	633 ± 64 b	628 ± 59 b	1517 ± 130 b	969 ± 120 b
	Leaf	1789 ± 275 b	401 ± 47 c	3334 ± 137 a	1237 ± 214 bc	417 ± 41 c	428 ± 45 c	1320 ± 131 bc	452 ± 59 c
Cd	Root	136 ± 21 a	8 ± 3 c	129 ± 3 a	21 ± 3 c	79 ± 4 b	7 ± 0.6 c	ND^a^	ND
	Stolon	34 ± 9 a	5 ± 1 c	27 ± 1 a	8 ± 1 c	17 ± 1 b	5 ± 0.2 c	ND	ND
	Stem	28 ± 6 a	4 ± 0.3 c	22 ± 1 a	6 ± 1 c	14 ± 0.5 b	2 ± 0.4 c	ND	ND
	Leaf	20 ± 4 a	3 ± 0.2 c	17 ± 0.5 a	5 ± 1 bc	10 ± 1 b	2 ± 0.1 c	ND	ND
Pb	Root	165 ± 44 b	54 ± 10 c	231 ± 30 a	65 ± 6 c	ND	ND	96 ± 20 c	28 ± 3 d
	Stolon	56 ± 8 a	29 ± 6 b	54 ± 6 a	38 ± 9 b	ND	ND	63 ± 15 a	21 ± 4 b
	Stem	50 ± 3 b	27 ± 1 bc	94 ± 2 a	46 ± 1 b	ND	ND	31 ± 1 bc	11 ± 1 c
	Leaf	39 ± 9 a	26 ± 2 b	40 ± 4 a	37 ± 5 a	ND	ND	27 ± 3 b	8 ± 2 c


*^a^ND means not detected. The data represent means ± standard error (SE, *n* = 5). Means in a column for a kind of metal followed by the same lower-case letter were not significantly different based on Fisher’s protected LSD test (*P* < 0.05). LY, ZZ, XT, and YY mean Liuyang, Zhuzhou, Xiangtan, and Yueyang City, respectively.*

### Microbial Community Functional Diversity

**FIGURE 1:**
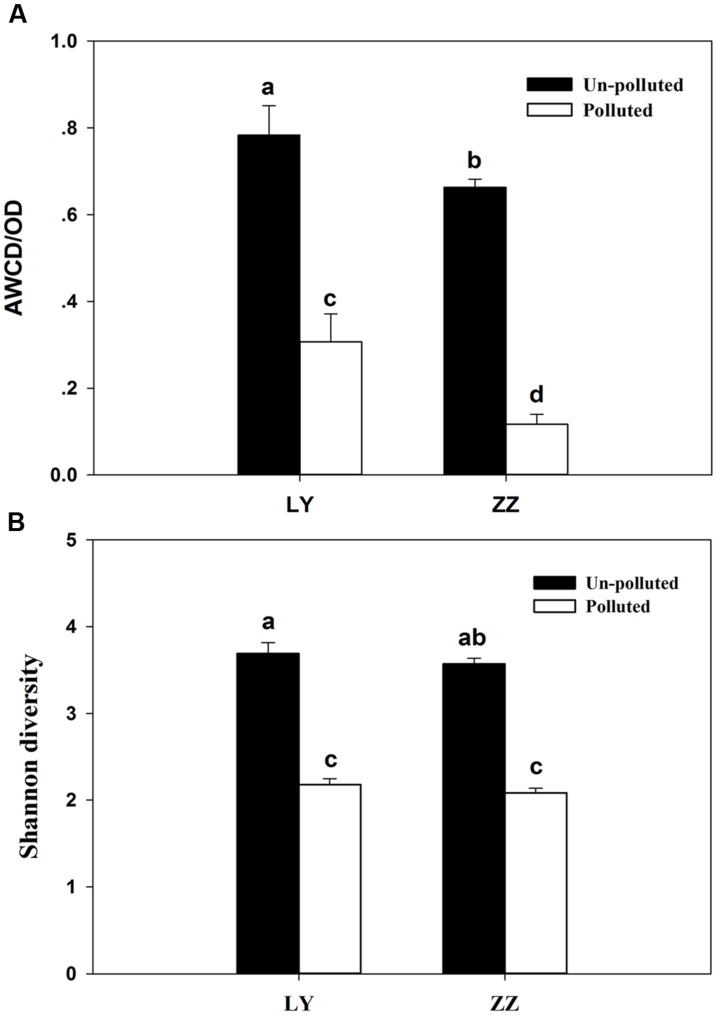
**Average well-color development (AWCD; **A)** and **(B)** Shannon diversity of soil microbial community.** LY and ZZ mean Liuyang and Zhuzhou City, respectively. Error bars are ±standard deviation (*n* = 5). Columns marked with the same lower-case letter were not significantly different based on Fisher’s protected LSD test (*P* < 0.05).

**FIGURE 2:**
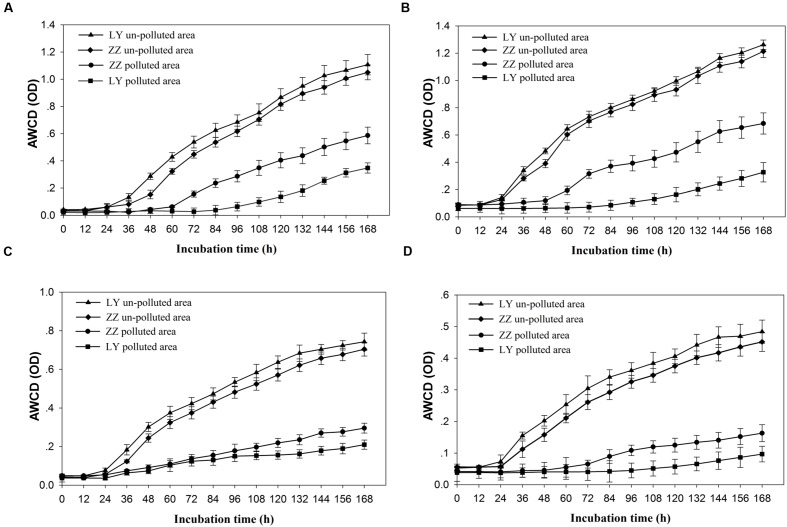
**Utilization of four kinds of carbon sources by soil microbial community from different sampling areas.** **(A–D)** Representing the amino acid and its derivates (AD), sugar and its derivates (SD), fatty acid and its derivates (FD), or secondary metabolites (SM), and average well color development (AWCD) indicating the average value of three replicated results of one Biolog-Ecoplate. LY and ZZ mean Liuyang and Zhuzhou City, respectively.

The relative capacity for substrate utilization in the different soils was as it appears in **Figure 1A**. Significant differences (*P* < 0.05) in the AWCD were found between the polluted and un-polluted soils. Polluted soils exhibited lower AWCD values than un-polluted soils. This result indicated that the substrate utilization rate by soil microbial communities being present in un-polluted soil was always higher than in polluted ones. The substrate utilization rate of soil microbial community present in the Zhuzhou samples was lower than that seen in those coming from Liuyang regardless of the pollution status. According to **Figure 1B**, the Shannon diversity was greater in un-polluted soil microbial communities than in polluted soil microbial communities. However, no difference in Shannon diversity was observed between Zhuzhou and Liuyang regardless of whether the soil was polluted or not. The utilization of carbon sources, divided into four categories (AD, SD, FD, and MD), began to increasing at 24 h incubation, regardless of the various sampling sites, with higher values always seen with un-polluted soil samples (**Figure 2**). There was no difference in the substrate utilization rates of above carbon source among the un-polluted soil. Higher SD and AD consumption levels were detected in microbial from Zhuzhou compared to those from Liuyang.

**FIGURE 3:**
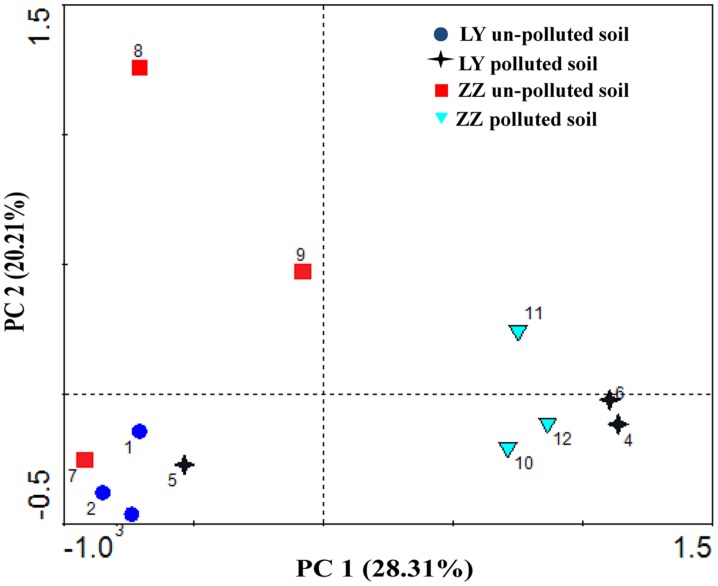
**Principal component analysis (PCA) for functional diversity of microbial communities in soil collected from different areas**.

Additionally, PCA was performed on the combined substrate utilization values from all sites (**Figure 3**). The first and second factors (PC1 and PC2) accounted for 28.31 and 20.21% of the variance, with a cumulative variance sum of 48.52%, respectively. The primary axis (PC1) mainly represented the soil types. PCA showed that both two polluted sites grouped well with each other and the un-polluted sites detached from the polluted sites. However, the values found with the analysis of the Liuyang site always clustered with those of the Zhuzhou site.

### Cadmium-Tolerance of Bermudagrass Accessions

The two tested factors (accession and sampling time) were significant for TQ, GR, Chl, and TR (**Table 3**). Interaction term involving the two tested factors was no significant for TQ, GR, Chl, and TR. Transpiration rate had a negative correlation with TQ, GR, and Chl, whereas TQ had a positive correlation with Chl and GR after 3-week’s Cd treatment (**Table 4**). Therefore, these four indicators are suitable for Cd-tolerance evaluation.

**Table 3:** Summary of analysis of variance for the effects of accessions, treatment time and the interactions on turf quality (TQ), chlorophyll content (Chl), transpiration rate (TR), and growth rate (GR).

Variables	TQ^a^ (0–9)	Chl (mg g^-1^ Fw)	TR (g day^-1^)	GR (mg day^-1^)
Accession (A)	^∗b^	^∗^	^∗^	^∗^
Time (T)	^∗^	^∗^	^∗^	^∗^
A^∗^T^d^	NS^c^	NS	NS	NS


*^a^Turf quality was assessed combined visual ratings of color intensity, leaf texture, and density. ^b∗^ indicates significant difference at *P* < 0.05. ^c^NS indicate no significant difference at *P* < 0.05. ^d^A^∗^T indicated the interaction effect of accessions and treatment on different index.*

**Table 4:** Correlation between TQ, chlorophyll content (Chl), TR, and GR for bermudagrass under cadmium stress.

	TQ	GR	Chl	TR
TQ	1			
GR	0.374^∗a^	1		
Chl	0.775^∗∗b^	0.227	1	
TR	-0.710^∗∗^	-0.473^∗^	-0.642^∗∗^	1


*^a∗^ means significant difference at *P* < 0.05. ^b∗∗^ means significant difference at *P* < 0.01.*

The subordinate function value of the four physiological traits was used to evaluate Cd-tolerance of different sites (**Table 5**). The subordinate function value of the four physiological traits in the four un-polluted sites was lower than that of the corresponding polluted sites. According to the average subordinate function value of the four traits, the trend of Cd-tolerance among the eight sites was as follows: Zhuzhou polluted site > Liuyang polluted site > Xiangtan polluted site > Zhuzhou un-polluted site > Yueyang polluted site > Liuyang un-polluted site > Yueyang un-polluted site > Xiangtan un-polluted site.

**Table 5:** The subordinate function of cadmium tolerance of bermudagrass collected from different areas of Hunan province.

Sampling sites	Soil type	Subordinate function value					Order
			
		TQ	Chl	TR	GR	Average	
Liuyang	Polluted	0.73	0.69	0.61	0.38	0.60	2
	Un-polluted	0.68	0.60	0.42	0.32	0.50	6
Zhuzhou	Polluted	0.68	0.60	0.57	0.67	0.67	1
	Un-polluted	0.57	0.54	0.36	0.53	0.53	4
Xiangtan	Polluted	0.66	0.57	0.33	0.57	0.57	3
	Un-polluted	0.47	0.29	0.19	0.39	0.39	8
Yueyang	Polluted	0.74	0.63	0.45	0.23	0.51	5
	Un-polluted	0.47	0.40	0.24	0.44	0.44	7


**TQ, Chl, TR, and GR mean turf quality, chlorophyll content, transpiration rate, and growth rate, respectively. The subordinate function value of each index of each accession was evaluated by the following equation: $X(\mu )=\frac{X-X_{\min}}{X_{\max}-X_{\min}}\,(1),$$X(\mu )=1-\frac{X-X_{\min}}{X_{\max}-X_{\min}}\,(2),$$\overset{¯}{X_{1}}=\frac{1}{n}\Sigma_{j=1}^{n}X_{ij}\,(3),$*X*(μ) is the subordinate function value of the μth indicator, *X* is the observed value of an indicator, *X*_*max*_ represents the maximum value of the indicator, and *X*_*min*_ is the minimum value. *X*_*1*_ is the mean of fuzzy subordinate function value, and *n* is the number of indicators. If an index was negatively correlated with Cd tolerance, membership value of Cd resistance was estimated by anti-membership function (formula 2). Otherwise it was calculated by formula 1. Mean value was counted following the accumulation of all membership values of Cd tolerance (formula 3).**

### Allelic Variation at SSR Loci and Comparison of Gene Diversity

The 46 pairs of primers used in the SSR analysis of 40 samples from the eight sites yielded 458 polymorphic bands. The number of markers per primer ranged from 3 to 16. Genetic diversity was estimated at each site as described previously (**Table 6**). The average gene diversity and PIC for the whole sample collected from polluted soil was 0.1833 and 0.1452, respectively, whereas the sample collected from un-polluted soil had values of 0.1690 and 0.1329, respectively. Interestingly, the genetic diversity and PIC of the Zhuzhou and Liuyang polluted site were higher than those of other locations. However, Yueyang polluted site had the lowest genetic diversity and PIC. The trend of gene diversity and PIC value within the eight sites was similar to the result of the Cd-tolerance evaluation using the subordinate function value.

**Table 6:** Genetic diversity within populations.

Soil type	Sampling sites	Group size	Average gene diversity	PIC^a^
Polluted	Liuyang	5	0.1944	0.1545
	Zhuzhou	5	0.1972	0.1562
	Xiangtan	5	0.1806	0.1431
	Yueyang	5	0.1608	0.1270
	Mean		0.1833	0.1452
Un-polluted	Liuyang	5	0.1762	0.1408
	Zhuzhou	5	0.1629	0.1291
	Xiangtan	5	0.1686	0.1287
	Yueyang	5	0.1687	0.1329
	Mean		0.1690	0.1329


*^a^Gene diversity and polymorphic information content (PIC) of populations were computed by the mean of pairwise differences among phenotypes of all members of the populations.*

### Cluster Analysis

**FIGURE 4:**
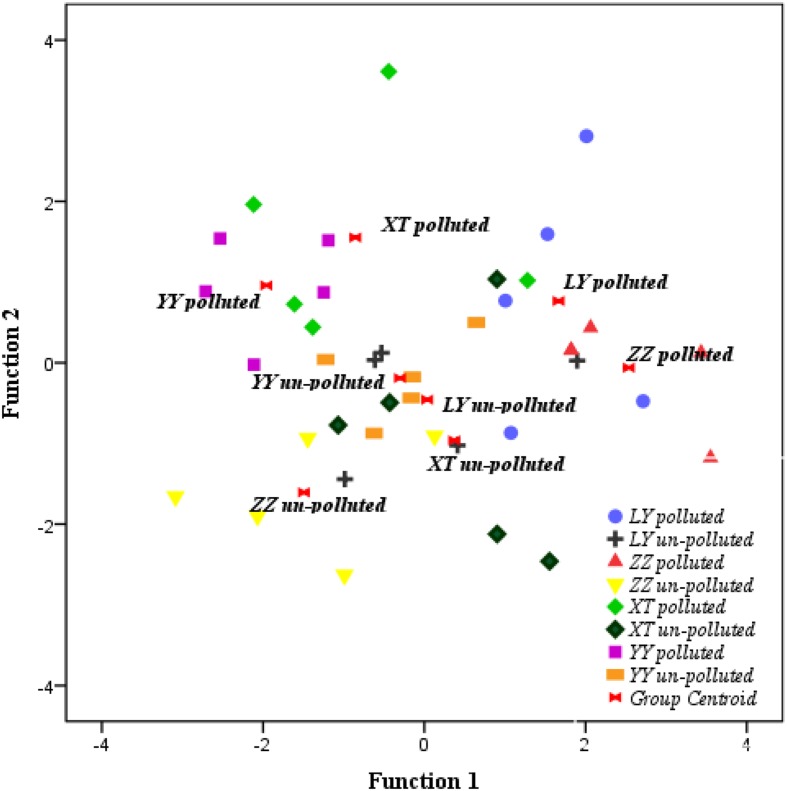
**Discriminant analysis of variations in phenotypic traits of 40 bermudagrass accessions under cadmium stress.** LY, ZZ, XT, and YY mean Liuyang, Zhuzhou, Xiangtan, and Yueyang City, respectively.

**FIGURE 5:**
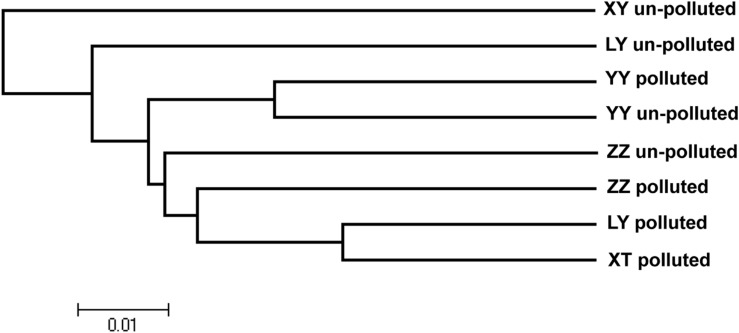
**Dendrogram of genetic relationships among populations.** The neighbor-joining method was applied to an average squared distance matrix among populations. Scale bar indicates average squared distance. LY, ZZ, XT, and YY mean Liuyang, Zhuzhou, Xiangtan, and Yueyang City, respectively.

Based on the discriminant analysis, 40 bermudagrass accessions clustered according to the similarity of physiological traits. Physiological cluster analysis showed that the majority of accessions from the un-polluted sites were grouped together (**Figure 4**). The accessions from the polluted sites were separated from those from the un-polluted sites. The neighbor-joining dendrogram (**Figure 5**) also showed a soil type pattern with polluted sites being clustered roughly according to their soil types, particularly for the Zhuzhou, Liuyang, and the Xiangtan group. Remarkably, the accessions from the Yueyang polluted soil areas were found distinct to the other groups from polluted sites.

## Discussion

Heavy-metal-contaminated soils impose various adverse effects on plant growth, such as plant macronutrient reduction, toxic concentrations of heavy metals, salinity, alkalinity, and poor physical structures (Bradshaw and Chadwick, 1982). In generally, soil phytotoxicity has been associated with 300 mg kg^-1^ total Zn (Lepp, 1981), 500 to 1000 mg kg^-1^ total Pb (Chaney, 1993) or 1 mg kg^-1^ total Cd (Peterson and Alloway, 1979). Therefore, the high total Zn and Cd of Liuyang, Zhuzhou, Xiangtan, and Yueyang soils might impose phytotoxic effects on higher plants (**Table 1**). However, our field investigation revealed that bermudagrass could thrive on those four polluted sites without any evident morphological, heavy metal-induced symptoms. This observation indicates that bermudagrass populations growing on the heavy metal contaminated sites could tolerate elevated metal concentrations. There is confirmation that heavy metal accumulation capacity varies widely among different varieties and species, and is affected by the levels of heavy metals in soil. Therefore, the varying quantities of heavy metals accumulated in bermudagrass growing on different sites reflected the levels of heavy metals in soil (**Table 2**). The results also indicate a close relationship between metal contents in plant tissues, and those in soils.

Heavy metal contamination not only results in adverse effects to plant yield and quality but also causes changes in the composition and activity of the soil microbial community (Giller et al., 1998b). Previous studies have demonstrated the adverse effect of heavy metals on soil microbial community structure and activity (Doelman, 1986; Yao et al., 2003). Knight and Liao pointed out that a slower rate of color development was observed with plants grown on heavy metal-polluted soils (Knight et al., 1997; Liao and Xie, 2007). In our study, the AWCD and the Shannon index values in the Biolog plates correlated with heavy metal concentrations. These results suggest that the total bioactivity, richness and diversity of microorganisms decreased with increasing heavy metal concentrations because microorganisms differ in their sensitivity to heavy metal toxicity. Heavy metals strongly reduced the numbers and species diversity of the soil microbial communities but, enhanced the development of metal-resistant microbial populations (Yao et al., 2003). In heavy metal polluted sites, the frequency of tolerant microbes increased with a rise in heavy metal concentrations (Kozdrój, 2001; Xie et al., 2014a). Results of the present study indicate that lower utilization rates of the four categories of (SD, AD, FD, and SM) carbon sources occurred in polluted sites. Strikingly, microbial community from Zhuzhou polluted soil samples showed higher SD and AD utilization rates than that from Liuyang polluted site. The Zhuzhou polluted site contained the highest concentrations of heavy metals. From these findings, we can assume that the Zhuzhou polluted area likely contained metal-resistant microbial community that could use carbon from sugar and amino acid.

It is factual that bermudagrass can withstand adverse edaphic condition including heavy metal toxicity (Wu and Antonovics, 1976; Xie et al., 2014b) and is widespread in the temperate and tropical areas. Therefore, bermudagrass bears a high potential for evolution of metal tolerance, and might be considered as useful in revegetation of heavy metal contaminated lands. It was necessary to evaluate its Cd-tolerance of bermudagrass to develop a type of bermudagrass suitable for phytoremediation. Previous studies indicated that heavy metals could cause severe phytotoxic effects, and might act as a driving force for the evolution of tolerant populations (Shu et al., 2002). Therefore, the identification of tolerant species from the natural vegetation of metalliferous mines is convenient (Baker and Proctor, 1990; Wu, 1990). In this study, the subordinate function value was applied to evaluate Cd-tolerance of bermudagrass at different sites. The higher subordinate function value in polluted bermudagrass populations confirms that these populations were able to withstand higher metal concentrations than the un-polluted populations, and might have evolved tolerance to Cd (**Table 5**). This observation is also in line with the view that the specific metal concentration in contaminated sites mainly governs the degree of tolerance (Bradshaw et al., 1965). The evolutionary processes of heavy metal tolerant populations have been noted in many hyperaccumulators, such as *Thlaspi caerulescens* and *Arabidopsis halleri* and in many tolerant plants, such as *Armeria maritima* and *Silene paradoxa* (Vekemans and Lefèbvre, 1997; Mengoni et al., 2000; Dubois et al., 2003; Pauwels et al., 2005). This observation supports the hypothesis that evolution of metal tolerance may be a major strategy for the potential of plant colonize contaminated areas. It has been shown that, microorganisms (bacteria and fungi) that can produce indole acetic acid (IAA), siderophores and 1-aminocyclopropane-1-carboxylate (ACC) deaminase are capable of stimulating plant growth and helping plants acquire sufficient iron for optimal growth (Burd et al., 2000). Cd contamination in soil is often associated with nutrient deficiency in a range of different botanical species because of their similar chemical properties (Ogo et al., 2014). Generally, microorganisms could protect plants grown in metal-contaminated soils by enhancing metal retention in roots and helping plants acquire sufficient nutrients and recycle the organic matter. Our result indicated that Zhuzhou polluted area likely contained metal-resistant microbial community, it might be one of the reasons why the bermudagrass growing on Zhuzhou polluted area was more Cd tolerant than others. Moreover, metal-resistant microbes have evolved various resistance and detoxification mechanisms and could be isolated and selected for their potential application in the bioremediation of contaminated sites. Therefore, it is very importance to characterize the metal-resistant microbial community and plants in sites like Zhuzhou in future works.

Investigations of the genetic variations among metal mining deposit populations and natural populations were carried out in many earlier studies, and some contradictory results surfaced. Previous studies on *S. paradoxa* and *Arrhenatherum elatius* indicated that the genetic diversity of populations from the contaminated and uncontaminated population were the same (Mengoni et al., 2000). A significant reduction in genetic diversity was also detected in populations from mining sites such as *Sedum alfredii* (Deng et al., 2007) and *Armeria maritima* (Vekemans and Lefèbvre, 1997). However, our results imply that the genetic diversity observed in populations from heavy metal contaminated sites was higher than those from populations from the neighboring heavy metal free areas. Although these outcomes do not meet theoretical expectations for a recent colonization event, similar results were reported previously for *Agrostis stolonifera* (Wu et al., 1975) and *Pinus sylvestris* L. (Prus-Glowacki et al., 1999). The same applies to *A. maritima* (Lefebvre and Kakes, 1981), *S. paradoxa* L. (Mengoni et al., 2000) and *S. maritime* (Baker and Dalby, 1980) for morphological characters, enzymatic variation and genetic diversity. Scholars consider metal-contaminated-areas to be ecological islands and expect the bottleneck to decrease the number of sensitive individuals while preserving that of the tolerant ones (Vekemans and Lefèbvre, 1997). However, a high number of tolerant individuals and successive colonization events in natural populations could reverse the initial genetic bottleneck attributable to selection (Mengoni et al., 2000). Pollen flow from adjacent normal populations could also be a possible source of variation. Also, Hunan is one of most severely heavy metal polluted provinces in China. Our preliminary field investigation revealed that in recent years, many restoration techniques have been applied in heavy metal polluted areas in the Hunan province, including phytoremediation approaches over there, and bermudagrass has been successfully used for repairing such contaminated soils. Human disturbance and environmental heterogeneity should also be considered as causes of genetic polymorphism (Gouyon et al., 1983).

Concerning the distribution of the genetic variation among the different populations, from the **Figure 5**, based on the polymorphic bands, the genetic variation among the different population could be seen. The result shows that neighbor-joining clustering is related to the geographical origin and soil contamination. These results show a clear separation between Yueyang and other populations growing at least 240 km apart. More interestingly, populations from Zhuzhou, Liuyang, and Xiangtan polluted sites were separated genetically from those from neighboring un-polluted sites. The same observation was vivid from the discriminant analysis conducted on phenotypic traits (TQ, GR, Chl, and TR). This observation indicated that edaphic conditions (i.e., heavy metals toxicity) might be more crucial for genetic variation of bermudagrass than geographic distance. In fact, similar results were also reported for several other tolerant plants, such as *A. maritima* (Vekemans and Lefèbvre, 1997), *S. paradoxa* (Mengoni et al., 2000), *S. alfredii* (Deng et al., 2007). The data from the Zhuzhou polluted population were unexpectedly not clustered with those of any other populations (**Figure 5**). The data from the field and hydroponic experiments indicates that the population from Zhuzhou polluted area possessed a superior tolerance than that of other polluted zones, and the concentrations of heavy metal for this site were the highest (**Tables 1** and **5**). The combined analysis of genetic variation with field and hydroponic experiments of the Zhuzhou polluted population suggest that the Zhuzhou polluted population might evolve into a novel ecotype with superior metal tolerance.

In summary, the Zhuzhou, Liuyang, Xiangtan, and Yueyang sites contained elevated concentrations of total Pb, Zn, and Cd, which would impose a high selection pressure for the evolution of metal-tolerant genotypes and ecotypes. The different quantities of heavy metals accumulated by bermudagrass growing on different sites reflect the levels of heavy metals in soil. The total bioactivity, richness, and diversity of microorganisms decreased with increasing heavy metal concentrations. The hydroponic experiments indicated that population of bermudagrass collected from polluted sites had evolved tolerance to Cd. This finding resonates with the view that specific metal concentration mostly governs the level of tolerance to heavy metals. Higher genetic diversity prevailed in populations from heavy metal-contaminated sites than in populations from heavy-metal-free sites, which might be attributable to a large number of tolerant individuals, successive colonization events, pollen flow from adjacent normal populations and human disturbance. The data from the field and hydroponic experiments indicate that heavy metal contamination seems to have a stronger effect on the genetic structure, and the population at the polluted Zhuzhou site might once evolve into a new ecotype possessing exceptional metal tolerance. The results of our study could be useful in the evaluation of soil quality and support efforts for the recovery of metal contaminated soils.

## Author Contributions

JF and LC designed the research. YX, JF, and WZ performed experiments and helped for data acquisition. YX wrote the manuscript in close collaboration with all authors. EA and YL reviewed the manuscript. All authors contributed to numerous discussions and revised the manuscript.

## Conflict of Interest Statement

The authors declare that the research was conducted in the absence of any commercial or financial relationships that could be construed as a potential conflict of interest.

## Footnotes

1 http://statgen.ncsu.edu/powermarker/index.html
