# Supplementary material for: Effect of Heavy Metals Pollution on Soil Microbial Diversity and Bermudagrass Genetic Variation
Source: Front Plant Sci. 2016 May 31;7:755. doi: 10.3389/fpls.2016.00755 (PMC4885870; doi:10.3389/fpls.2016.00755)
Supplement: Supplementary file 1 [file Table_1.DOCX]

Supplementary Material

**Effect of heavy metals pollution on soil microbial diversity and bermudagrass** **genetic variation**

**Yan Xie^1^, Jibiao Fan^1, 2^, Weixi Zhu^1^, Erick Amombo^1^, Yanhong Lou^1, 3^, Liang Chen ^1^*, Jinmin Fu ^1^***

^1^ *Key Laboratory of Plant Germplasm Enhancement and Specialty Agriculture,* *Wuhan Botanical Garden, Chinese Academy of Sciences, Wuhan City, Hubei, P.R. China*

^2^ *Graduate University of Chinese Academy of Sciences, Beijing 100049, P.R. China*

*^3^College of Resources and Environment, Shandong Agricultural University, Tai'an, P.R. China*

^*^Corresponding author. Telephone: +86 027 87511506;

^*^ Email: chenliang1034@126.com

[jfu@wbgcas.cn](mailto:jfu@wbgcas.cn)

Table S1 Characteristics of 46 SSR primers used for the genetic relationship analysis

| PP No. | Primer name | Forward primer | Loci | Number of allele | Size range |
| --- | --- | --- | --- | --- | --- |
| 1 | CTCA1-505  CTCA1-506 | TCCAATATAACACCCCCACA TGGTGATGAACGACACCTTT | (AC)7 | 7 | 195-292 |
| 2 | CTCA1-517  CTCA1-518 | GTTGCAAAAGGGGTGTTTCT  ATAAGCCCAAGTGGATCACC | (AC)5-(AC)12 | 8 | 153-206 |
| 3 | CTCA1-541  CTCA1-542 | CGTGCCCTAAGTTCAGTTCA  TTCTCATGACTTAGCACCCG | (GT)14 | 14 | 257-340 |
| 4 | CTCA1-553  CTCA1-554 | CCGAGAAGTTTCCTTTCCTG  AGGCACATTGATGACCAAGA | (TG)18 | 13 | 166-236 |
| 5 | CTCA1-559  CTCA1-560 | ACCTTTGTGATGGATTGGGT  CTTGCATAATGATGCCACCT | (CA)14 | 10 | 85-190 |
| 6 | CTCA2-575  CTCA2-576 | TTTAGACCCTGGATCCGAAC  ATAAGCTTCCGCTTGACAGG | (TAG)5-(AC)14 | 14 | 304-363 |
| 7 | CTCA2-593  CTCA2-594 | TGTGGAAATCTATCAGCCCA GCTTCCCATCCCACTGTACT | (CA)15 | 8 | 196-233 |
| 8 | CTCA3-645  CTCA3-646 | TCTGTTGGGCTTTGTTCTTG  ACATGTAGTCTGCGGATGGA | (AC)5-(AC)21 | 6 | 221-238 |
| 9 | CTCA3-659  CTCA3-660 | CAAGGCTACCTTTTGTCAAGG  GGTACAAGAAAGCCTCTGCC | (GT)21 | 9 | 149-229 |
| 10 | CTCA3-671  CTCA3-672 | TCTGTCTGGCCAAAAACTTG  TTTGATTGCTACTGCCAAGC | (TG)25 | 15 | 258-287 |
| 11 | CTCA3-703  CTCA3-704 | CAACCTGTGGCATTGAAGTT  AATTAGGGAGGGTAAGGCGT | (GT)30 | 14 | 227-271 |
| 12 | CTCA3-711  CTCA3-712 | CCAATTCCCATAATCCCAAC  GACTCAGCGCAATTGTTGTT | (GA)6 | 10 | 253-347 |
| 13 | CTCA3-715  CTCA3-716 | TTGGCTCCATCCAATCACTA  TCCAGCCTGACATCCAAATA | (TG)6 | 9 | 160-182 |
| 14 | CTCA4-741  CTCA4-742 | GCTCTCTACAGGGTGCATCA  TAAAAGAATGCCCTGTGTCG | (TG)20-(TG)12 | 8 | 214-282 |
| 15 | CTCA4-743  CTCA4-744 | TAGCGCTTTTGAAACGACAC  GAATCCCATCACTGCCTTTT | (CA)20 | 3 | 177-240 |
| 16 | CTCA4-761  CTCA4-762 | ATGGACTGCAGACATTGGTC  CTTGAAGATATGGTGCTCCG | (TG)18 | 4 | 242-271 |
| 17 | CTCA4-767  CTCA4-768 | TATATAGCCCGCCATGAACA  AGCCATCCACAGGTTCATTT | (GT)23-(GA)7 | 9 | 195-230 |
| 18 | CTCA4-781  CTCA4-782 | GCCACATCCGTATTTCTTGA  GGAGACGCTATGTGTTGGTG | (CA)23 | 11 | 275-300 |
| 19 | CTCA4-789  CTCA4-790 | GTATAAGATGAGCTCGGCCC  TAGCAGAAGCAGCATCCATC | (TC)16-(AC)15 | 9 | 204-258 |
| 20 | CTCA5-813  CTCA5-814 | GGAGATGTCCAGCTTGATGA  CTCGTACTACCCCACCCAGT | (TG)12 | 9 | 258-286 |
| 21 | CTCA5-849  CTCA5-850 | CCAGATCTCTTTCGCCAACT  ACTCCACTGAAAGCCATTCC | (AC)7-(CA)8 | 13 | 173-194 |
| 22 | CTCA6-867  CTCA6-868 | ATGTCGATTTCCAACGTCCT  GGATCTTTTGCATGTCGCTA | (AC)10 | 13 | 95-163 |
| 23 | CTCA6-885  CTCA6-886 | ATGGGTCTTGTCATGGGAAT  CCTGTTTGCCTCCCATACTC | (TG)11-(AG)9 | 6 | 146-214 |
| 24 | CTCA7-943  CTCA7-944 | CGACGTGTGACAAACAAGAA  GCATGCTTGTCAGCAGTTTT | (AC)23 | 12 | 192-245 |
| 25 | CTCA7-977  CTCA7-978 | TGGTGAAAGAAACTGCAACC  ACAGGGTGCATCAACACAAT | (CA)31 | 5 | 113-195 |
| 26 | CTCA7-1027  CTCA7-1028 | CAGACAGAGAGTGCCAAGGA  AGAACCGGTTGCACTTTCAT | (TC)17-(AC)18 | 8 | 329-362 |
| 27 | CTCA8-1069  CTCA8-1070 | TGATAGCGAGACTCCTGTGG  TTTTGCCTTCACAACCGTAG | (GT)8-(TG)9 | 16 | 257-294 |
| 28 | CTCA8-1073  CTCA8-1074 | GTTGAGCCTAGACCCGCTAC  TCAGGGTGACACACACACAC | (TG)17-(GT)11 | 6 | 156-250 |
| 29 | CTCONT-11  CTCONT-12 | AACAGTATTGCTCCAGCCAG  CGGTGCGTAACACCATTAAC | (CA)5 | 7 | 232-284 |
| 30 | CTCONT-13  CTCONT-14 | ATGGAAAGTCCAAGTGGTCA  TTGCAAGTGGCAGATTCAAG | (TC)5 | 13 | 98-154 |
| 31 | CTCONT-65  CTCONT-66 | GTTGACGCGAGAAACAAAGA  TACATCTGCAGTTACCCACG | (TG)18 | 11 | 119-190 |
| 32 | CTCONT-109  CTCONT-110 | AACGACTGAACCTTCCGAGT  TTTGTGTGTCTGTGTGTGGG | (AC)10-(CA)5  -(CT)7 | 15 | 297-340 |
| 33 | CTCONT-123  CTCONT-124 | CAGGCTCTACACGACCTCAA  AAAGGCTGGCAATAAACCAC | (AC)14 | 8 | 110-160 |
| 34 | CTGA1-1199  CTGA1-1200 | ATAAAAGGGCAACCGACTTG  ATCGAGCAACCCTAGCAATC | (TC)21 | 14 | 151-187 |
| 35 | CTGA2-1341  CTGA2-1342 | ACAACATCAACTTCCGTTGC  TGCTAGCTGCCAGTGTCTCT | (GA)21 | 13 | 175-232 |
| 36 | CTGA3-1417  CTGA3-1418 | ATGCTCATCAGCAGGAACAG  AGCTTACCAATCCGCAAAGT | (GA)26 | 8 | 260-324 |
| 37 | CTGA4-1543  CTGA4-1544 | ACCCTTTTTACACTCGGTCG  CATTGTAGCGCTTCCTTCAA | (AG)15 | 11 | 263-379 |
| 38 | CTGA7-1949  CTGA7-1950 | GTTAGGGCTTCATCCCATGT  AGCAAGCATCTTGGTGTCAG | (CT)13 | 9 | 103-141 |
| 39 | CTGA7-1985  CTGA7-1986 | GCAGAGCAAAAGAGCCTACC  CCAAAAGCCACCCTACACTT | (GA)27 | 10 | 205-230 |
| 40 | CTGA8-2109  CTGA8-2110 | CTACTGCTGAAAACCTTGCG  ACCGTCAACAACGATAACGA | (AG)32 | 12 | 169-218 |
| 41 | CTGA8-2163  CTGA8-2164 | AAGGGGCATATCTCTTGGTG  CACATGGAAGACCACCATTC | (GA)33 | 11 | 169-248 |
| 426 | CTAAG1-2219  CTAAG1-2220 | AAACTCATTAGGTTGCCGGT  CAGAATGGAGTTCGGGACTT | (AGA)5-(AAG)5 | 3 | 267-326 |
| 43 | CTCONT-295  CTCONT-296 | TGGAAAGGAGAGGAATCTGG  TACGTAACCGAGCTCCTCCT | (GAA)6-(AGG)7 | 8 | 420-458 |
| 44 | CTCONT-343  CTCONT-344 | CTCGTGAACCTTGGGGTACT  GTCTCCCTGGCTGGTATGTT | (GAA)8 | 11 | 234-279 |
| 45 | CTAAT1-2513  CTAAT1-2514 | GGAGCGAGAGAGAGAAGGAA  CGTACGTGTGTCCCTCATTC | (TAT)12 | 11 | 280-325 |
| 46 | CTAAT2-2583  CTAAT2-2584 | GTACTGGGGGAGGTTTGAGA  TGCAGTGTGCACTATGGAAA | (AAT)9 | 14 | 175-256 |
